# Socioeconomic and health disparities in adults diagnosed with type 1 diabetes mellitus before age 18: insights from the Italian PASSI surveillance system

**DOI:** 10.3389/fpubh.2025.1655035

**Published:** 2025-09-16

**Authors:** Giulia Zamagni, Valentina Minardi, Maria Masocco, Federica Asta, Riccardo Candido, Gianluca Tornese, Giulia Bresciani, Valentina Manfredini, Elena Frattolin, Claudia Veronica Carletti, Daniela Germano, Eleonora Maurel, Luca Ronfani, Lorenzo Monasta

**Affiliations:** ^1^Institute for Maternal and Child Health—IRCCS “Burlo Garofolo”, Trieste, Italy; ^2^National Center for Disease Prevention and Health Promotion, Italian National Institute of Health, Rome, Italy; ^3^Department of Medical Surgical and Health Sciences, University of Trieste, Trieste, Italy; ^4^Friuli-Venezia Giulia Regional Coordination of Associations of People Living with Diabetes, Trieste, Italy; ^5^Friuli-Venezia Giulia Regional Center PASSI Surveillance System—ASUGI, Trieste, Italy

**Keywords:** public health, socioeconomic inequalities, health inequalites, type 1 diabetes mellitus, quality of life

## Abstract

**Background:**

Type 1 diabetes mellitus is a lifelong condition with consequences that extend well beyond glycaemic control, often impacting individuals' socioeconomic status and overall quality of life. In Italy, the broader effects of early-onset type 1 diabetes on social and health-related outcomes have been insufficiently investigated. Therefore, this study aimed to investigate the socioeconomic impacts of type 1 diabetes among adults diagnosed with the condition before age 18.

**Methods:**

Using data from the Italian Behavioral Risk Factor Surveillance System (PASSI) collected between 2011 and 2018 and in 2023, we analyzed key outcomes in adults aged 18–50 who were diagnosed with type 1 diabetes before age 18 and were on insulin therapy. Each case was matched by age and sex to two non-diabetic controls. Descriptive statistics and multivariable logistic regression were used to compare key indicators.

**Results:**

Our sample included 993 participants (331 cases and 662 controls). Cases had significantly higher odds of being unemployed [OR = 1.57 (1.20–2.07)], experiencing severe financial difficulties [OR = 1.81 (1.05–3.13)], and reporting poor self-rated health [OR = 6.64 (2.53–17.43)]. Cases also had an increased likelihood of reporting physical impairment for 1–13 days [OR = 1.91 (1.30–2.81)] and ≥14 days [OR = 2.95 (1.54–5.65)], mental health impairment for 1–13 days [OR = 2.16 (1.46–3.19)], and daily activity limitations for 1–13 days [OR = 1.73 (1.06–2.82)].

**Conclusions:**

These findings highlight the multifaceted burden of type 1 diabetes and the need for integrated approaches to care that address not only clinical but also socioeconomic and psychosocial dimensions of the disease.

## Introduction

Type 1 diabetes mellitus is a chronic autoimmune disease characterized by the destruction of pancreatic beta cells, leading to absolute insulin deficiency and lifelong dependence on exogenous insulin therapy (1). Despite advancements in diabetes care, including the development of insulin analogs, continuous glucose monitoring, and automated insulin delivery systems, type 1 diabetes remains a condition that imposes significant medical, psychological, and socioeconomic burdens on affected individuals.

Over the years, the prevalence of type 1 diabetes has been increasing globally, with 8.4 million individuals living with the condition in 2021, and ~500,000 new cases being diagnosed. Moreover, projections suggest that by 2040, the number of individuals with type 1 diabetes will rise to between 13.5 and 17.4 million, highlighting the growing public health challenge posed by this disease (2).

However, the epidemiology of type 1 diabetes varies widely across different regions and demographic groups. Incidence rates are highest in Northern Europe, where they reach up to 60 per 100,000 individuals annually, whereas in regions such as East Asia and Latin America, the incidence remains below 3 per 100,000 per year (3). These differences underscore the importance of region-specific public health initiatives, including improved access to diagnostic tools, early intervention strategies, and equitable distribution of diabetes management resources (4–7).

While medical advancements have significantly improved life expectancy and quality of life for individuals with type 1 diabetes, the persistent disparities in health and socioeconomic outcomes remain a critical concern, as individuals with type 1 diabetes often face barriers that extend beyond glycaemic management, including higher risk of unemployment, financial hardship, and lower educational attainment compared to their non-diabetic peers (6). In addition, the financial burden of managing type 1 diabetes, including costs related to medications, glucose monitoring, and dietary requirements, can contribute to economic instability and stress (8).

Given these challenges, it is essential to understand the broader impact of type 1 diabetes on socioeconomic and health-related outcomes to inform targeted policy interventions and healthcare strategies aimed at reducing these disparities.

This study seeks to examine differences in marital status, employment, financial situation, self-perceived health, and physical and mental health limitations among adults diagnosed with type 1 diabetes before the age of 18 and matched controls in Italy. The findings will provide deeper insights into the long-term impacts of T1DM and help inform the development of targeted interventions aimed at improving the quality of life and overall well-being of affected individuals.

## Methods

This study was conducted within the framework of the Joint Action on Cardiovascular Diseases and Diabetes (JACARDI), under Work Package 11, which focuses on the labor participation of people living with CVD or diabetes (https://jacardi.eu/services/labour-participation-of-people-living-with-cvd-or-dm/).

### Data source and study population

This study used data from PASSI (*Progressi delle Aziende Sanitarie per la Salute in Italia*), the *Italian Behavioral Risk Factor Surveillance System* (BRFSS), over the years 2011–2018 and 2023.

PASSI is a cross-sectional surveillance, through sample surveys, and since 2008 it has been continuously monitoring the prevalence of the major behavioral risk factors for chronic non-communicable diseases and compliance level to the main preventive measures among the adult population (18–69 years of age) residing in Italy. It covers several topics, out of which smoking habits, physical inactivity, excess weight, alcohol consumption, the state of physical and psychological well-being, and some aspects relating to the quality of life connected to health. It represents a useful tool to plan health promotion and prevention interventions and monitor their effectiveness over time.

PASSI is inspired by the BRFSS international framework of the US Centers for Disease Prevention and Control (CDC) (https://www.cdc.gov/brfss/index.html) and it is mandated by the Italian Ministry of Health and officially acknowledged under the Decree of the Prime Minister's Office on Registries and Surveillances, 3 March 2017 (9). The National Institute of Health in Italy, known as Istituto Superiore di Sanità, is responsible for coordinating this surveillance at the national level, but data collection is carried out by Local Health Units (LHUs), coordinated by regions, through sample surveys.

In each LHU participating, monthly random samples are extracted from the enrolment list of residents stratified by sex and age (18–34, 35–49 and 50–69 years) proportionally to the size of the respective strata in the general population. Specially trained personnel from the LHUs' public health departments administer telephone interviews using a standardized questionnaire. Eligibility criteria include being within the target age range, having a reachable phone number (landline or mobile), not being hospitalized or institutionalized, understanding the Italian language (in the autonomous province of Bolzano the interviewees have the option of being interviewed in German), and having the ability to participate in the interview. Collected data are anonymised and stored in a national database. Details about methodological issues have been described elsewhere (10, 11).

### Indicator definitions

A case of type 1 diabetes was classified as a person who declared to have been diagnosed by a physician for diabetes mellitus, declared an age at diagnosis < 18 years, and to be undergoing insulin therapy.

A control case was defined as a person who reported no physician diagnosis of non-communicable diseases among those investigated in the PASSI questionnaire (kidney failure, chronic bronchitis, emphysema, respiratory failure, bronchial asthma, stroke or cerebral ischemia, diabetes, myocardial infarction, ischemic heart or coronary artery disease, other heart diseases, cancers including leukemia and lymphoma, chronic liver disease or cirrhosis).

Socio-demographic characteristics, behavioral and health indicators of interest for the analyses were: age, sex, citizenship (Italian, foreign), marital status (married, single, widowed, separated/divorced), educational attainment (never attended school, primary school degree, secondary school degree, high school degree, Bachelor's degree or more), economic difficulties (very easily, easily, with some difficulty and with a lot of difficulties in managing monthly income), employment status (employed, looking for a job and inactive), sedentary lifestyle, Body Mass Index (BMI) (normal weight: 18.5 ≤ BMI ≤ 24.9 kg/m^2^, overweight: 25 ≤ BMI ≤ 29.9 kg/m^2^ and obesity: BMI ≥ 30 kg/m^2^), self-reported health (very good, good, discrete, poor, very poor health), self-reported unhealthy days due to physically problems (0 days, 1–13 days and >=14 days), self-reported unhealthy days due to psychological problems (0 days, 1–13 days and >=14 days), self-reported days with limitation in daily activity, due to physically or mental health (0 days, 1–13 days and >=14 days), depressive symptoms by Patient Health Questionnaire-2 (PHQ-2) (12) (Supplementary Table 1).

### Statistical analysis

In our study, we used PASSI data collected in the period 2011–2018 and 2023, selecting cases aged 18–50 years who had been diagnosed DM before the age of 18 and undergoing insulin therapy (as a proxy of T1DM cases) and matched them on age and sex to a double number of controls, using data collected. Specifically, we included all available cases identified during the study period and applied exact matching. Controls were defined as individuals without any reported diagnosis of chronic disease before the age of 18. Given this study design, survey weights were not applied in the analysis. Data for the years 2019–2022 were not used because the age at DM diagnosis had not been collected.

Key outcomes of interest for case-control comparison were marital status, educational level, economic difficulties, self-reported health, and self-reported unhealthy days (physical, mental and activity impairment).

Categorical variables were presented as counts and percentages, while continuous variables were expressed as either mean and standard deviation (SD) or median and interquartile range (IQR), based on the results of the Shapiro-Wilk normality test. Comparisons between categorical variables were performed using the Chi-square test or, when appropriate, Fisher's exact test. Continuous variables were compared using the Student's *t*-test or the Wilcoxon Mann-Whitney test, based on data distribution.

The association between type 1 diabetes and the key outcomes was evaluated via univariate and multivariable logistic regression (binary or multinomial). In the multivariable analysis, the effect of type 1 diabetes on key outcomes was adjusted for age and sex to account for potential confounding effects (13). All analyses followed a complete-case approach. Statistical significance was set at a *p*-value < 0.05. All analyses were conducted using Stata 18 and R software.

## Results

**Figure 1:**
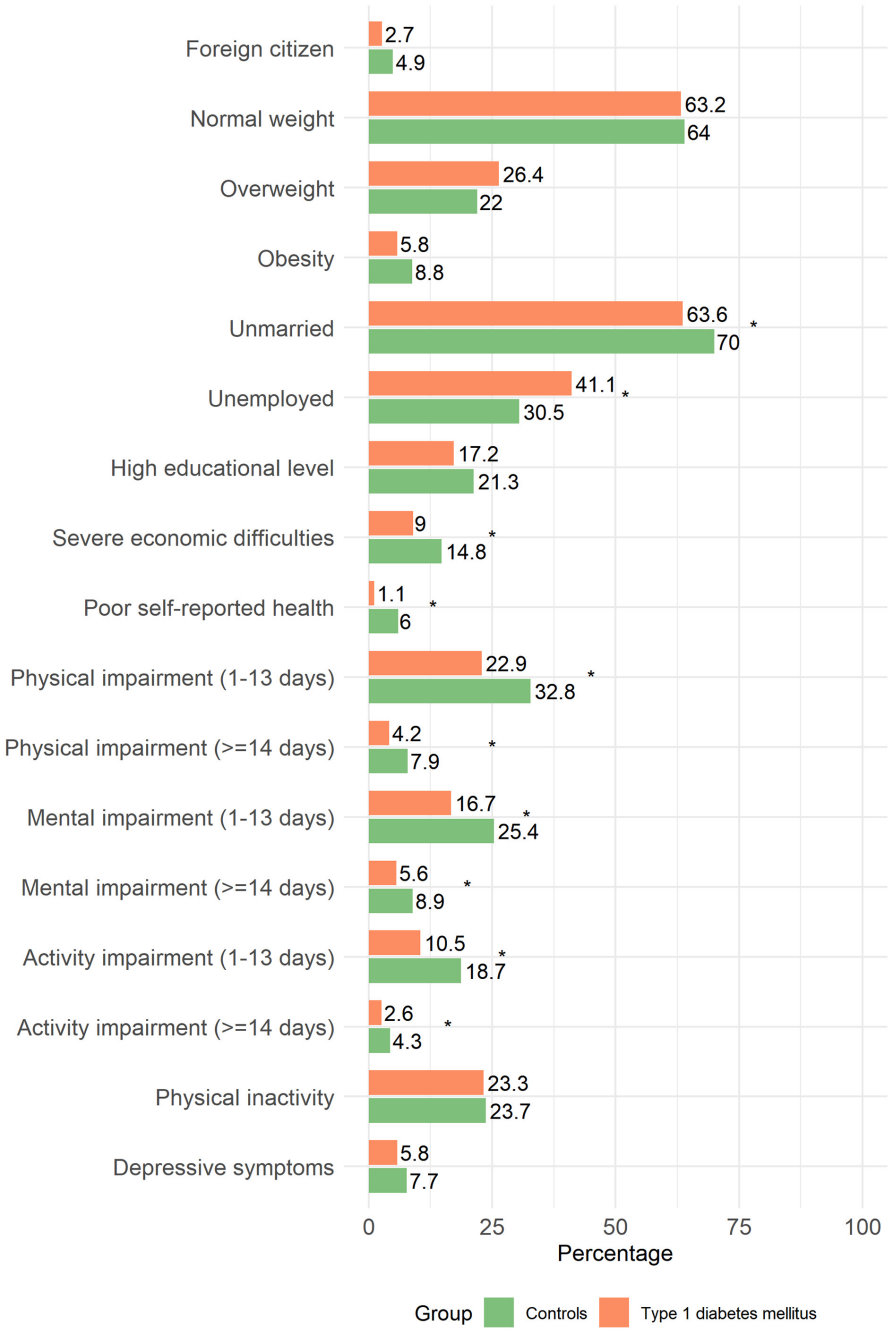
Frequency distribution of respondents' characteristics by group (controls vs. type 1 diabetes mellitus).

From 2011 to 2018 and 2023, 319,801 people aged 18–69 residing in 90% of the Italian LHUs were interviewed. Among them, 331 18–50-year-olds were identified as type 1 diabetes cases according to the given definition. In the study, a total of 993 individuals were included: 331 individuals with type 1 diabetes (i.e., cases) and 662 healthy controls. Data was complete for most variables included in the analyses. Only a few indicators had small amounts of missing data, with overall completeness remaining very high (i.e., >96% across all variables and within each group). Specifically, information was available for physical impairment in 979 participants (329 cases and 650 controls), mental impairment in 972 (327 cases and 645 controls, and activity impairment in 975 (327 cases and 648 controls). In addition, data were available for BMI in 988 participants (329 cases and 659 controls), citizenship in 985 (328 cases and 657 controls), depressive symptoms in 961 (323 cases and 638 controls), and physical inactivity in 986 (329 cases and 657 controls), out of the total sample of 993 individuals. The mean age of the considered population was 32.6 years (SD = 0.6) for the cases and 32.5 years (SD = 0.4) for the controls. Females were 47% in both groups. As shown in Figure 1, most participants were Italian citizens (95% in the control group and 97% in the group with type 1 diabetes). BMI was comparable across groups, with most participants reporting a normal weight (63% in cases and 64% in controls). Significant differences emerged in marital status, as individuals with type 1 diabetes were more likely to be unmarried than controls (70% vs. 64%, *p* = 0.046). While educational attainment was similar between groups, unemployment was significantly higher in the group with type 1 diabetes (41% vs. 31%, *p* = 0.001). Consistently, economic hardship was reported more frequently among cases, with 14.8% experiencing severe financial difficulties compared to 9% of controls (*p* = 0.020). Self-reported health also varied significantly between groups: 6% of individuals with type 1 diabetes rated their health as “*poor/very poor*”, compared to 1% of controls (*p* < 0.001). Regarding physical impairment (unhealthy days for physical health) in the past 30 days, 8% of cases and 4% of controls reported feeling unwell for ≥14 days, while 33% of cases and 23% of controls reported limitations for 1–13 days (*p* < 0.001). On average, cases had 3 days (SD = 0.4) of compromised physical health compared to 2 days (SD = 0.2) for controls. Impairment due to poor mental health (unhealthy days for psychological problems) was also significantly higher in the group with type 1 diabetes. Specifically, 9% of cases and 6% of controls reported impairment for ≥14 days, while 25% of cases and 17% of controls reported limitations for 1–13 days (*p* < 0.001). Cases had on average 3 days (SD = 0.4) of compromised mental health compared to 2 days (SD = 0.2) for controls. Similarly, 4% of cases and 3% of controls reported being unable to carry out daily activities for ≥14 days due to poor physical or mental health status, while 19% of cases and 10% of controls reported limitations for 1–13 days (*p* < 0.001). On average, controls reported 1 day (SD = 0.2) of impaired daily activity per month, while individuals with type 1 diabetes reported 2 days (SD = 0.3). No significant differences were observed between the two groups in terms of sedentary lifestyle or the proportion of interviewed declaring depressive symptoms, although this proportion was slightly higher for the cases. Among individuals with type 1 diabetes, 90% were under the care of a Diabetes Center, with 59% receiving exclusive follow-up from the center and 31% being jointly monitored by their family physician and the Diabetes Center. On average, cases reported 3 visits (SD = 0.3) to their family physician for diabetes-related consultations and 3 visits (SD = 0.2) to the Diabetes Center in the past 12 months.

At univariate analysis, type 1 diabetes emerged as a significant risk factor for various socioeconomic and health-related conditions (Table 1). Specifically, cases had a higher risk of being unmarried [OR = 1.34 (1.01–1.78)], unemployed [OR = 1.59 (1.21–2.09)], and experiencing severe financial difficulties (OR = 1.83 (1.20–2.79)]. In addition, type 1 diabetes was strongly associated with poor self-reported health status [OR = 5.19 (3.83–7.04)]. The risk of experiencing 1–13 days of impaired physical health per month was nearly twice as high in cases compared to controls [OR = 1.76 (1.31–2.37)]. The risk was even higher if we consider ≥14 days of impaired physical health [OR = 2.34 (1.33–4.11)]. A similar association was found for mental health, with an increased risk of 1–13 days of impairment [OR = 1.79 (1.29–2.49)] and ≥14 days of impairment [OR = 1.88 (1.12–3.14)] for cases compared to controls. While the risk of ≥14 days of daily activity impairment was not significantly different between the two groups, cases still had twice the risk of experiencing 1–13 days of limitations per month compared to controls [OR = 2.00 (1.38–2.92)].

**Table 1:** Results of the univariate logistic regression assessing the association between type 1 diabetes mellitus and key socioeconomic and health-related outcomes.

**Outcome**	**OR for type 1 diabetes mellitus**	**95% CI**	***p*-value**
Unmarried (ref. married)	1.34	1.01; 1.78	0.042
Unemployed (ref. employed)	1.59	1.21; 2.09	0.001
Poor self-reported health (ref. discrete/good health)	5.19	3.83; 7.04	<0.001
High educational level (degree or higher) (ref. other)	1.30	0.93; 1.83	0.129
Mild financial difficulties (ref. none)	1.11	0.83; 1.47	0.476
Severe financial difficulties (ref. none)	1.83	1.20; 2.79	0.006
Physical impairment (1–13 days) (ref. 0 days)	1.76	1.31; 2.37	<0.001
Physical impairment (>=14 days) ref. (0 days)	2.34	1.33; 4.11	0.004
Mental impairment (1–13 days) ref. (0 days)	1.79	1.29; 2.49	<0.001
Mental impairment (>=14 days) ref. (0 days)	1.88	1.12; 3.14	0.019
Activity impairment (1–13 days) ref. (0 days)	2.00	1.38; 2.92	<0.001
Activity impairment (>=14 days) ref. (0 days)	1.84	0.89; 3.79	0.106

OR, odds ratio; CI, confidence interval.

Subgroup analyses examining the effect of age group and sex on impairment within cases and controls separately are presented in Supplementary Tables 2A, B respectively. The findings indicate that being 35–50 years old was not significantly associated with any of the outcomes examined in either cases or controls. However, among controls, significant sex differences were found. Specifically, female controls had higher odds of physical impairment for 1–13 days per month compared to males [OR = 1.76 (1.09–2.83)]. They also showed an increased risk of mental impairment over the same period [OR = 1.65 (1.08–2.50)] and had even higher odds of daily activity impairment [OR = 2.24 (1.26–3.98)] compared to males. Among cases, females were significantly more likely to experience physical impairment than males for >=14 days per month [OR = 2.49 (1.10–5.66)], while no sex differences were observed for the remaining outcomes.

**Figure 2:**
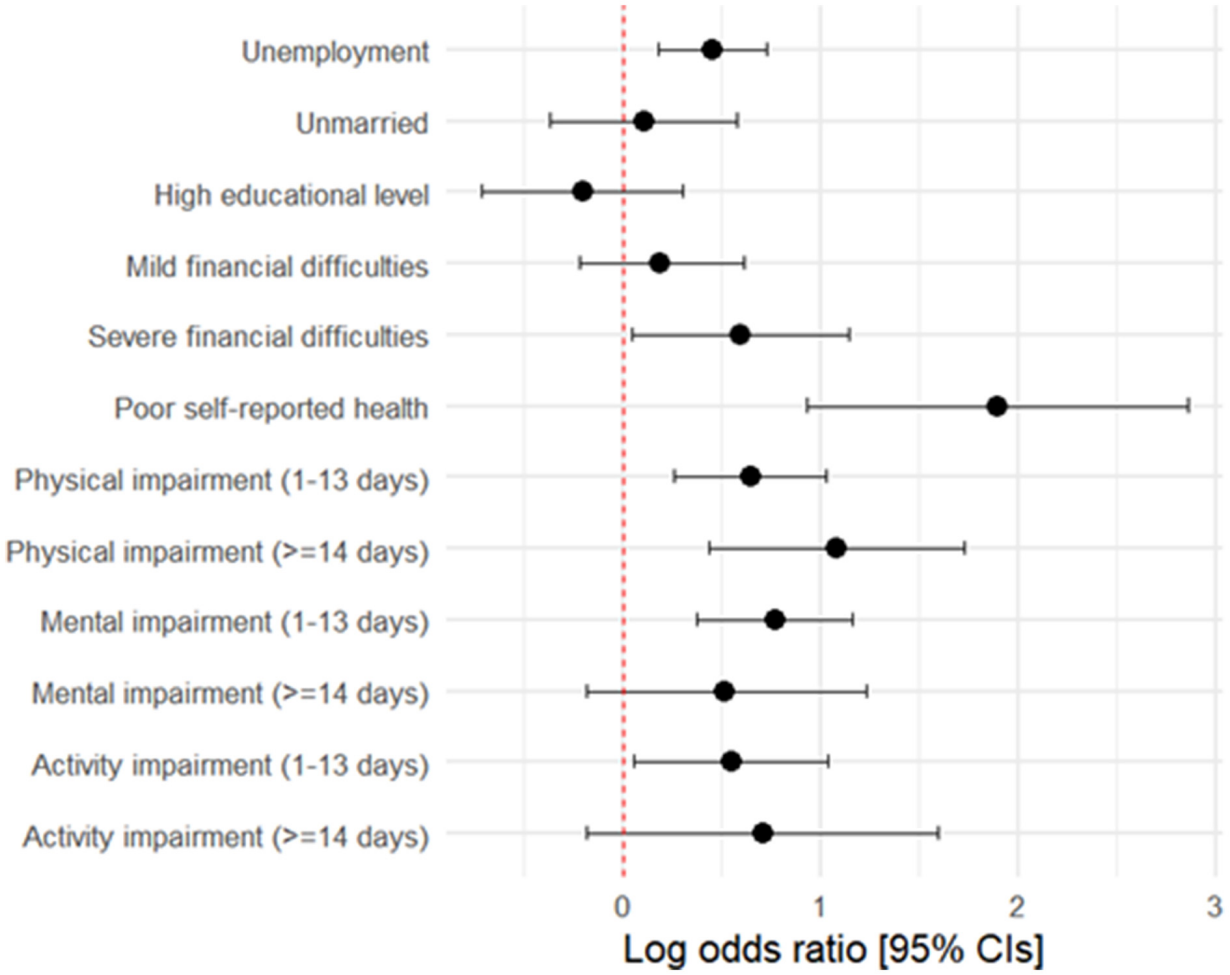
Log-odds ratios and 95% CIs for the association between type 2 diabetes mellitus and key outcomes, adjusted for sex and age.

After adjusting for age and sex, type 1 diabetes persisted as a significant risk factor for unemployment [OR = 1.57 (1.20–2.07)] and severe financial difficulties [OR = 1.81 (1.05–3.13)], while the association with marital status lost significance (Figure 2). Analogously, the risk of poor self-reported health status remained significantly higher in the presence of type 1 diabetes [OR = 6.64 (2.53–17.43)]. Regarding physical health, females showed an increased risk of experiencing 1–13 days of impairment per month compared to males [OR = 1.69 (1.13–2.52)], as did individuals aged 35–50 years [OR = 1.54 (1.03–2.32)] and cases [OR = 1.91 (1.30–2.81)]. However, for ≥14 days of physical impairment, type 1 diabetes remained a significant risk factor [OR = 2.95 (1.54–5.65)], while sex and age lost significance. For mental health, females had a higher risk of 1–13 days of impairment per month [OR = 1.53 (1.05–2.24)], as well as individuals with type 1 diabetes [OR = 2.16 (1.46–3.19)]. No significant associations were found with mental impairment for ≥14 days per month. A similar trend was observed for daily activities, in which the risk of 1–13 days of impairment was higher for both females [OR = 1.80 (1.07–3.02)] and individuals with type 1 diabetes [OR = 1.73 (1.06–2.82)], while no significant associations were found between age, sex or type 1 diabetes and impaired daily activities for ≥14 days per month.

## Discussion

Our study offers valuable insight into the socioeconomic and health-related disparities faced by individuals with type 1 diabetes in Italy. In particular, our results indicate that people diagnosed with type 1 diabetes before the age of 18 are at significantly higher risk of unemployment, financial hardship and poor self-perceived health status compared to the control group (that is, peers who declared not having any of the chronic non-communicable disease investigated by PASSI). Interestingly, while type 1 diabetes was significantly associated with economic and health disadvantages, there were no significant differences in educational attainment between individuals with type 1 diabetes and controls, likely reflecting the efforts made in recent years to ensure that children and adolescents with type 1 diabetes can attend school safely and without restrictions (14). Nevertheless, despite similar levels of education, individuals with type 1 diabetes continue to face barriers to employment and financial stability, which may contribute to long-term socioeconomic disparities.

This aligns with the available literature indicating that the burden of type 1 diabetes extends beyond medical management and involves multiple aspects of daily life, potentially undermining overall well-being (15, 16). In Denmark, individuals with type 1 diabetes are more frequently unemployed and on sick leave, and evidence from a systematic review indicates that those diagnosed in childhood face persistent challenges in employment despite achieving comparable academic outcomes to peers without diabetes, suggesting that labor market participation may represent a more sensitive indicator of social disadvantage than educational attainment (17, 18).

These findings underscore the urgent need for targeted policies aimed at improving institutional and financial support for individuals with chronic conditions, particularly those diagnosed early in life, to reduce socioeconomic inequalities over the life course. Several initiatives illustrate ongoing attempts to provide more comprehensive support for people living with type 1 diabetes beyond clinical care. For instance, in the United Kingdom, Diabetes UK has developed specific workplace guidance, such as the booklet “Supporting Someone with Diabetes at Work”, which provides practical advice for employers and colleagues to promote inclusion and ensure that people with diabetes are supported in managing their condition during working hours (19). For children and young people, the England National Health Service has also launched DigiBete (http://www.digibete.org), a digital platform offering videos and educational resources designed to empower families and improve self-management of type 1 diabetes. In Italy, the national association FAND (Federazione Associazioni Nazionali Diabetici) promotes awareness-raising initiatives and organizes educational courses aimed at supporting people with diabetes and their families, fostering empowerment and advocacy in the community (https://www.fand.it/). Together, these examples highlight the value of comprehensive strategies that integrate workplace support, digital education, and community engagement to improve quality of life and long-term outcomes for people living with type 1 diabetes.

Our results further emphasize the substantial impact of type 1 diabetes on self-perceived health status and physical and mental well-being. Individuals with type 1 diabetes reported more days with physical and mental impairments, as well as greater limitations in daily activities, than the control group. The compromised well-being of individuals with type 1 diabetes is well documented in the scientific literature, with studies consistently reporting an elevated risk of psychiatric comorbidities among children and adolescents, including mood and anxiety disorders, eating disorders, substance use, and even suicidal behavior (20–22).

These findings highlight the importance of integrating mental health support into diabetes care programs and ensuring their accessibility, as psychological distress and diabetes-related burnout can significantly affect disease management and overall quality of life (23, 24).

For example, Versloot and colleagues demonstrated that implementing an integrated stepped-care model within an interprofessional pediatric diabetes clinic can improve adolescents' overall quality of life and diabetes-related quality of life (25). Moreover, a consensus document signed by the American Diabetes Association (ADA) and the European Association for the Study of Diabetes (EASD) recommends the systematic assessment and periodic monitoring of emotional health, at least annually, to promote early case finding, enhance emotional well-being, and improve patient satisfaction with care (26). Furthermore, another position document of the AD states that diabetes teams should include a mental health professional to provide direct support and advise the clinical team (27).

Despite the advancements in diabetes technology aimed at improving glycaemic control, insulin pump usage remains particularly low in Italy, especially among adults (28). This finding contrasts with the fact that 90% of individuals with type 1 diabetes are followed by a Diabetes Center, suggesting that barriers to accessing these technologies are not primarily due to limited clinical coverage. In Italy, insulin pumps (both conventional and patch-pumps) are reimbursed by the NHS for all individuals with insulin-treated diabetes mellitus (29). Although this policy ensures that patients have access to necessary diabetes management devices without direct costs, the actual uptake of insulin pump therapy remains limited despite the growing evidence supporting their positive impact on the overall quality of life, including improved glycaemic control and increased flexibility in daily diabetes management (30–32). Therefore, it is crucial to identify and overcome the barriers that limit access to insulin pumps. Strategies may include targeted educational activities to raise awareness about the benefits of their use, training programs for healthcare professionals to better support patients in considering this option and addressing psychological factors that may deter individuals from transitioning to insulin pump use. However, we were not able to evaluate insulin pump use or potential barriers to its adoption in our study, as the PASSI questionnaire does not include specific questions on this aspect.

Our subgroup analysis revealed notable sex disparities among the control group, with females showing higher odds of experiencing physical, mental, and daily activity impairments for 1 to 13 days per month compared to males. Hormonal differences, the higher prevalence of autoimmune comorbidities among females, and potential differences in healthcare-seeking behaviors may contribute to this persistent disparity (33, 34). However, among individuals with type 1 diabetes, these differences were not observed, except for females having higher odds of being physically impaired for 14 or more days per month compared to males. This suggests that the challenges introduced by type 1 diabetes may attenuate the known sex differences in health-related impairments, despite some gaps remaining, suggesting that sex-specific factors continue to play a role in disease burden.

While our results provide valuable insights, some limitations must be acknowledged. First, this study relies on self-reported data, which may be subject to misclassification, particularly regarding financial difficulties, employment status, and self-perceived health. Second, we did not assess regional disparities in healthcare access, which may play a significant role in shaping health outcomes for individuals with type 1 diabetes. Third, the small sample size in some subgroup analyses may have limited the statistical power to detect subtle differences. Fourth, certain specific aspects—such as number of children and insulin pump use—could not be thoroughly assessed due to the retrospective nature of the analysis and the use of a pre-defined questionnaire.

## Conclusions

Our findings emphasize the need for comprehensive healthcare strategies that extend beyond glycaemic management to address the broader social and economic challenges associated with type 1 diabetes. Policies that provide financial assistance, workplace support, and integrated mental health care could play a crucial role in improving long-term outcomes for individuals with type 1 diabetes.

Future research should assess the effectiveness of such interventions and explore additional determinants, including healthcare access and social support networks, that may influence the socioeconomic and health trajectories of individuals with type 1 diabetes. Furthermore, an emphasis on disparities among Italian regions could provide a more nuanced understanding of how local healthcare policies and resources impact health outcomes and quality of life in this population.

## Data Availability

The data analyzed in this study is subject to the following licenses/restrictions: The data used in this study are drawn from the Italian PASSI surveillance system, which is managed by the Istituto Superiore di Sanità. Due to confidentiality agreements and institutional regulations, the raw data cannot be shared publicly. Requests for access can be directed to the corresponding author, who will liaise with the data custodians as appropriate. Requests to access these datasets should be directed to Valentina Minardi, valentina.minardi@iss.it; Maria Masocco, maria.masocco@iss.it; Federica Asta, federica.asta@iss.it.
