# Correction: Socioeconomic and health disparities in adults diagnosed with type 1 diabetes mellitus before age 18: insights from the Italian PASSI surveillance system

**DOI:** 10.3389/fpubh.2025.1715382

**Published:** 2025-10-14

**Authors:** Giulia Zamagni, Valentina Minardi, Maria Masocco, Federica Asta, Riccardo Candido, Gianluca Tornese, Giulia Bresciani, Valentina Manfredini, Elena Frattolin, Claudia Veronica Carletti, Daniela Germano, Eleonora Maurel, Luca Ronfani, Lorenzo Monasta

**Affiliations:** ^1^Institute for Maternal and Child Health—IRCCS “Burlo Garofolo”, Trieste, Italy; ^2^National Center for Disease Prevention and Health Promotion, Italian National Institute of Health, Rome, Italy; ^3^Department of Medical Surgical and Health Sciences, University of Trieste, Trieste, Italy; ^4^Friuli-Venezia Giulia Regional Coordination of Associations of People Living with Diabetes, Trieste, Italy; ^5^Friuli-Venezia Giulia Regional Center PASSI Surveillance System—ASUGI, Trieste, Italy

**Keywords:** public health, socioeconomic inequalities, health inequalites, type 1 diabetes mellitus, quality of life

In the original version of this article, the affiliation number 1 was incorrectly listed as:

Institute for Maternal and Child Health—Burlo Garofolo, Trieste, Italy.

The correct affiliation is:

Institute for Maternal and Child Health—IRCCS “Burlo Garofolo”, Trieste, Italy.

The original version of this article has been updated.

