# Supplementary material for: Socioeconomic and health disparities in adults diagnosed with type 1 diabetes mellitus before age 18: insights from the Italian PASSI surveillance system
Source: Front Public Health. 2025 Sep 16;13:1655035. doi: 10.3389/fpubh.2025.1655035 (PMC12479510; doi:10.3389/fpubh.2025.1655035)
Supplement: Supplementary file 1 [file Table_1.docx]

**Supplementary Table 1.** Definitions of PASSI indicators.

| **Dimension** | **Indicator** | **Question** | **Answers** | **Outcome definition and coding** |
| --- | --- | --- | --- | --- |
| **Self-reported health status** | Diabetes | Has a doctor, nurse, or other health professional ever told you that you had diabetes? | yes vs no | Prevalence of individuals who report to have been diagnosed with diabetes by a physician |
|  | Self-reported health | Would you say  that in general  your health  is… | 1. Very Good 2. Good 3. Discrete 4. Poor 5. Very poor | Poor: poor or very poor  Discrete: Discrete  Good: good or very good |
|  | Physical impairment | Now thinking about your physical health, which includes physical illness and injury, for how many days during the past 30 days was your physical health not good? | number of days | 0 days vs 1-13 days vs >=14 days |
|  | Mental impairment | Now thinking about your mental health, which includes stress, depression, and problems with emotions, for how many days during the past 30 days was your mental health not good? | number of days | 0 days vs 1-13 days vs >=14 days |
|  | Activity impairment | During the past 30 days, for about how many days did poor physical or mental health keep you from doing your usual activities, such as self-care, work, or recreation? | number of days | 0 days vs 1-13 days vs >=14 days |
|  | Depression (PHQ2 test) | Over the last 2 weeks, how often have you been bothered by little interest or pleasure in doing things?  AND  Over the last 2 weeks, how often have you been bothered by feeling down, depressed, or hopeless? | number of days | A score is assigned basing on the duration of symptoms in days; if the overall score is ≥ 3, the person is considered to be suffering from depressive symptoms |
| **Physical Activity** | Physical inactivity | Physical inactive people are identified as those do not perform any vigorous or moderate-intensity aerobic physical activity during leisure time (such as running, bicycling, brisk walking, vacuuming) and do not perform regular jobs that requires considerable physical effort | yes vs no | Prevalence of individuals who resulted to be physically inactive |
| **Obesity and overweight** | Body Mass Index | Can you tell me your height without shoes?  AND  Can you tell me your weight, without clothes, shoes or with light clothes? | BMI= Weight (kg)/Height^2^(cm) | Normal weight: 18.5≤BMI≤24.9 kg/m^2  Overweight:  25≤ BMI≤29.9 kg/m^2  Obesity:  BMI≥30 kg/m^2 |
| **Sociodemographic characteristics** | Marital status | Which is your current marital status? | 1. Married 2. Single 3. Widowed 4. Separated/Divorced | Married vs others |
|  | Citizenship | What is you citizenship? | 1. Italian 2. Others 3. Dual citizenship (Italian and other) | Italian vs others |
|  | Educational level | What is your highest educational level? | 1. Never attended school 2. Primary school degree 3. Secondary school degree 4. High school degree 5. Bachelor’s degree or more | High educational level (Bachelor’s degree or higher) vs others |
|  | Economic difficulties | With the financial resources available to you (from your own income or family support), how do you manage to get through the month? | 1. Very easily 2. Quite easily 3. With some difficulty 4. With a lot of difficulty | Very easily/easily vs with some difficulty vs with a lot of difficulties |
|  | Unemployment | At this moment, are you: employed, looking for a job, or inactive? |  | Employed vs looking for a job or inactive |
| **Diabetes care management*** | Who primarily manages your diabetes care? | | 1. Family doctor 2. Both family doctor and diabetes center 3. Diabetes center 4. Other health provider 5. None | Family doctor vs diabetes center vs both vs other |
|  | In the past 12 months, how many times have you consulted your family doctor for a diabetes check-up? | | Number of days | Mean |
|  | In the past 12 months, how many times have you visited the diabetes center for a diabetes check-up? | | Number of days | Mean |

*Only for T1DM respondents

**Supplementary Table 2A.** Odds Ratios (ORs) and 95% Confidence Intervals (Cis) for age group and key outcomes.

| **Outcome*** | **Group** | **OR for 35-50 years** [95% CI]** | **p-value** |
| --- | --- | --- | --- |
| **Mental impairment (1-13 days)** | **Cases** | 1.55 [0.93-2.58] | 0.093 |
| **Mental impairment (1-13 days)** | **Controls** | 0.97 [0.64-1.48] | 0.914 |
| **Mental impairment (>=14 days)** | **Cases** | 0.76 [0.34-1.71] | 0.551 |
| **Mental impairment (>=14 days)** | **Controls** | 1.52 [0.77-3.00] | 0.227 |
| **Physical impairment (1-13 days)** | **Cases** | 1.50 [0.94-2.43] | 0.091 |
| **Physical impairment (1-13 days)** | **Controls** | 1.31 [0.91-1.90] | 0.155 |
| **Physical impairment (>=14 days)** | **Cases** | 1.34 [0.59; 3.05] | 0.526 |
| **Physical impairment (>=14 days)** | **Controls** | 1.55 [0.71-3.38] | 0.316 |
| **Activity impairment (1-13 days)** | **Cases** | 1.57 [0.89-2.75] | 0.116 |
| **Activity impairment (1-13 days)** | **Controls** | 1.06 [0.64-1.76] | 0.897 |
| **Activity impairment (>=14 days)** | **Cases** | 0.79 [0.26-2.43] | 0.784 |
| **Activity impairment (>=14 days)** | **Controls** | 1.20 [0.45-3.15] | 0.805 |

*Reference category: 0 days; **Reference category: 18-34 years

**Supplementary Table 2B.** Odds Ratios (ORs) and 95% Confidence Intervals (CIs) for sex and key outcomes.

| **Outcome** | **Group** | **OR [95% CI]** | **p-value** |
| --- | --- | --- | --- |
| **Mental impairment (1-13 days)** | **Cases** | 1.46 [0.88-2.44] | 0.154 |
| **Mental impairment (1-13 days)** | **Controls** | 1.64 [1.08-2.50] | ***0.025*** |
| **Mental impairment (>=14 days)** | **Cases** | 1.93 [0.88-4.24] | 0.113 |
| **Mental impairment (>=14 days)** | **Controls** | 1.77 [0.89-3.52] | 0.118 |
| **Physical impairment (1-13 days)** | **Cases** | 1.33 [0.92-1.93] | 0.132 |
| **Physical impairment (1-13 days)** | **Controls** | 1.76 [1.09-2.83] | ***0.022*** |
| **Physical impairment (>=14 days)** | **Cases** | 2.49 [1.10-5.66] | ***0.028*** |
| **Physical impairment (>=14 days)** | **Controls** | 1.21 [0.53-2.74] | 0.676 |
| **Activity impairment (1-13 days)** | **Cases** | 1.64 [0.98-2.72] | 0.071 |
| **Activity impairment (1-13 days)** | **Controls** | 2.24 [1.26-3.98] | ***0.006*** |
| **Activity impairment (>=14 days)** | **Cases** | 1.77 [0.64-4.63] | 0.325 |
| **Activity impairment (>=14 days)** | **Controls** | 1.36 [0.46-3.98] | 0.591 |

*Reference category: 0 days; **Reference category: males
